# Internal ribosome entry site of bFGF is the target of thalidomide for IMiDs development in multiple myeloma

**DOI:** 10.18632/genesandcancer.11

**Published:** 2014-03

**Authors:** I-Chia Lien, Lin-Yea Horng, Pei-Lun Hsu, Chia-Ling Wu, Hui-Ching Sung, Rong-Tsun Wu

**Affiliations:** ^1^ Institute of Biopharmaceutical Sciences, National Yang-Ming University, Taipei, ROC (Taiwan); ^2^ Research Centre for Drug Discovery, National Yang-Ming University, Taipei, ROC (Taiwan)

**Keywords:** Thalidomide, Internal ribosome entry site (IRES), bFGF

## Abstract

Although new analogues of immunomodulatory drugs (IMiDs) are being developed for MM, the molecular mechanism of these drugs remains unclear. In the current study, we used MM cell lines as a model to investigate the molecular mechanism of thalidomide and to compare its potency with IMiDs such as pomalidomide. We determined that thalidomide did not inhibit cell proliferation of RPMI8226 and U266 MM cells, whereas pomalidomide showed a significant inhibitory effect on these two MM cell lines. Interestingly, we further demonstrated that although thalidomide down-regulated bFGF translation through the inhibition of IRES even at 0.1 μg/ml, pomalidomide did not have a similar affect bFGF levels. A colony formation assay demonstrated that thalidomide and the bFGF knock-down clones caused a significant reduction in the clonogenic ability of MM cells, and treatment with exogenous bFGF can recover the clonogenic ability of thalidomide-treated cells and knock-down clones, but not that of pomalidomide-treated cells. This implies that thalidomide, but not pomalidomide, targets the IRES of FGF-2.

In conclusion, our results highlight a non-cytotoxic anticancer drug target for thalidomide, the IRES of bFGF, and provide the mechanistic rationale for developing IMiDs as anti-cancer therapeutics in MM patients, with improved potency and fewer side effects.

## INTRODUCTION

Thalidomide, an anti-angiogenic drug approved by the FDA in 1998 for the treatment of Erythema nodosum leprosum (ENL) [1], has been demonstrated to have anti-cancer properties [2] and was used as a novel anti-myeloma drug for relapsed and refractory disease in 1999 [2,3]. To date, multiple myeloma (MM) remains largely incurable [4]. Although originally marketed in Europe as a sedative and antiemetic, reports of teratogenic effects of thalidomide led to its withdrawal from the market in 1961[5]. The teratogenic activity of thalidomide was proposed to be mediated by its binding to both the DNA and RNA of the fetus, when administered either p.o. or i.p. Consequently, binding of the glutarimide moiety of thalidomide to DNA might alter the secondary structure of DNA [6, 7].

Thalidomide was the first chemotherapeutic drug to demonstrate anticancer activity against myeloma in more than 30 years [8], although the precise mechanisms of action of the drug remain undefined. Thalidomide analogs (IMiDs) are hypothesized to act through multiple mechanisms [9], Lenalidomide has a better toxicity profile than thalidomide, and pomalidomide may overcome resistance to lenalidomide [10]. A correlation between high plasma basic fibroblast growth factor (bFGF) levels and a positive response to thalidomide treatment in MM has also been demonstrated [11].

bFGF is a growth factor that exists as several isoforms that differ in their N-terminal extensions, subcellular distribution and function [12, 13]. The expression of bFGF transcripts is under the control of a G-rich promoter. Our previous report showed that thalidomide down-regulates bFGF transcription and translation by targeting its G-rich promoter [14]. The smallest low molecular weight (LMW) variant, an 18 kDa bFGF, is released by cells and acts through the activation of cell surface FGF-receptors, whereas the high molecular weight (HMW) (22, 22.5, 24 and 34 kDa) bFGFs localize to the nucleus [12] and signal independently of FGFR [15]. Despite significant evidence documenting the expression and intracellular trafficking of HMW bFGF, many important questions remain about the physiological or pathological roles and mechanisms of action of HMW bFGF [16]. The nuclear-targeted HMW 24 kDa bFGF may induce specific cell functions through intracrine mechanisms. The effect of nuclear bFGF on the metastatic potential of carcinoma cells demonstrated spontaneous metastasis *in vitro* and *in vivo* [17, 18]. Interestingly, our data showed that thalidomide regulates the cellular distribution of bFGF in glioma cells [14] and provides evidence for the role of the HMW bFGF isoform in carcinoma. Vacca *et al.* showed that the plasma levels of bFGF were significantly higher in patients with active disease than in those with non-active MM and MGUS [19, 20].

Anchorage-independent growth (AIG) is a hallmark of cancer cells [21, 22], and colony formation assay in soft agar was used as a simple selection method for human tumor stem cells [21]. Since Hamburger and S. E. Salmon demonstrated a low percentage of clonogenic cells in the bulk tumor mass prompted a search for the CSC in MM [21], colony-forming unit/clonogenic assays in methylcellulose or soft agar were commonly used to identify the MM stem cells [23]. Our previous data have shown that the AIG of U-87 glioma cells was suppressed by thalidomide, and knocking down or even down-regulating bFGF expression is sufficient to decrease the tumor growth *in vivo* [14]. The ability to exhibit anchorage-independent cell growth is considered a fundamental property of cancer cells because it has been correlated with tumor cell aggressiveness *in vivo*, such as tumorigenic and metastatic potential [24].

The majority of cellular stresses have lead to the inhibition of cap-dependent translation [25]. Functional studies have demonstrated that cellular IRES (internal ribosome entry site) -mediated translation prevails under conditions when cap-dependent translation is compromised, such as hypoxia, heat shock, irradiation, apoptosis and tumorigenesis. Nevertheless, the precise roles of IRES-dependent translation under physiological and pathological conditions remain to be determined. Some mRNAs, however, are translated by a cap-independent mechanism, which is mediated by ribosome binding to internal ribosome entry site (IRES) elements located in the 5’-untranslated region. These mRNAs code for products that include growth factors such as bFGF and VEGF, which are required for growth [26, 27]. Some reports suggested the increased expression of several proteins that are under IRES control, such as oncogenes and growth factors involved in the progression of cancer [28]. However, the role of the bFGF IRES in tumorigenesis has yet to be fully documented [29, 30]. Elucidating the mechanism of IRES-mediated translation and its regulation will be a major challenge in this field [31].

In the present study, we examined the role of IRES-mediated regulation of FGF-2 translation in tumorigenesis as a critical step not only in solid tumors but also in multiple myeloma. Using thalidomide as a tool, we elucidate its molecular mechanism in MM, compare the developed IMiDs such as pomalidomide, and highlight the right direction for developing more potent IMiDs with fewer side effects for MM patients.

## RESULTS

### Distinct Effects of Thalidomide and Pomalidomide on bFGF, VEGF and IL-6 Expression in RPMI8226 and U266 Cells

**Figure 1:**
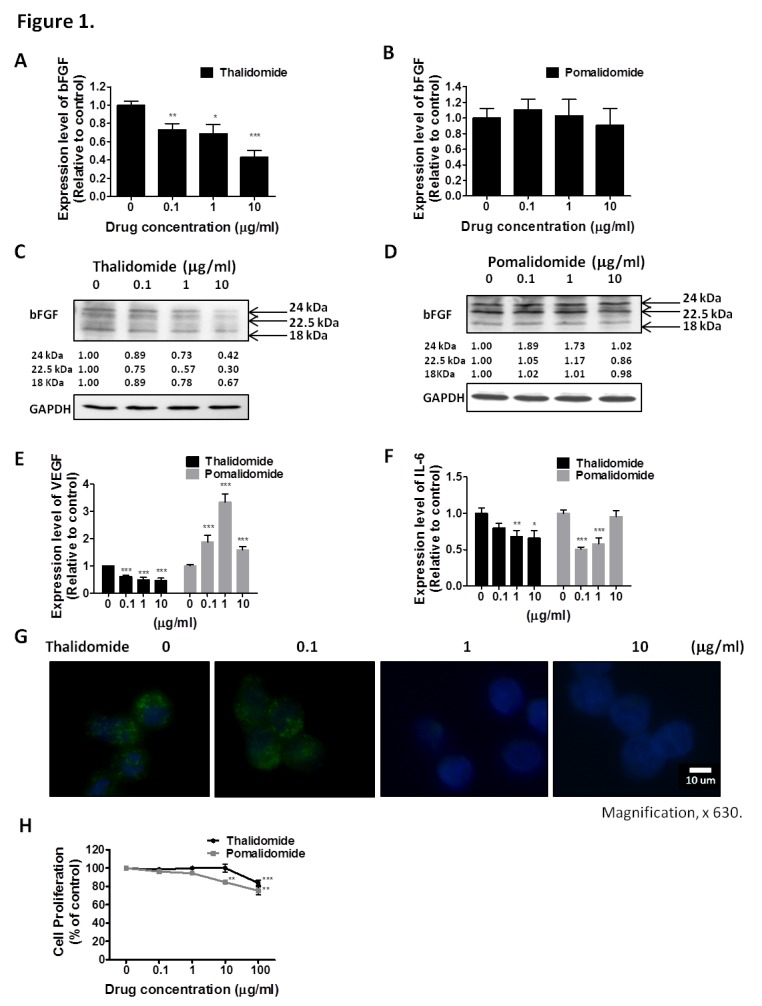
Effect of thalidomide or pomalidomide on bFGF, VEGF and IL-6 expression in RPMI8226 cells. RPMI8226 cells were treated with (A) thalidomide or (B) pomalidomide for 4 hours. The bFGF mRNA expression was monitored by real-time PCR, with the expression of GAPDH used as an internal control. Protein extracts from RPMI8226 cells treated with (C) thalidomide or (D) pomalidomide for 4 hours were subjected to Western blot for analysis of bFGF protein level. GAPDH is the internal control. RPMI8226 cells were treated with thalidomide and pomalidomide for 4 hours and mRNA levels of (E) VEGF and (F) IL-6 were monitored by real-time PCR. (G) Thalidomide regulates bFGF expression and cellular distribution in RPMI8226 cells. Immunofluorescence detection of bFGF in RPMI8226 cells treated with thalidomide for 4 h. Cellular distribution of bFGF was studied by fluorescence microscopy. DNA was stained with H33258 as a nuclear marker. Magnification, × 630. (H) Viable cell counts using trypan blue. Cell viability of RPMI8226 cells treated with thalidomide and pomalidomide. RPMI8226 cells, which were treated with the indicated concentrations (0, 0.1, 1 and 10 μg/mL) of thalidomide and pomalidomide for 72 hours, were stained with trypan blue. Data were collected from at least 3 independent experiments. The results were expressed as the relative index of untreated control ± S.E.M. (*, P < 0.05, **, P < 0.01, ***, P < 0.001, Student's t-test).

**Figure 2:**
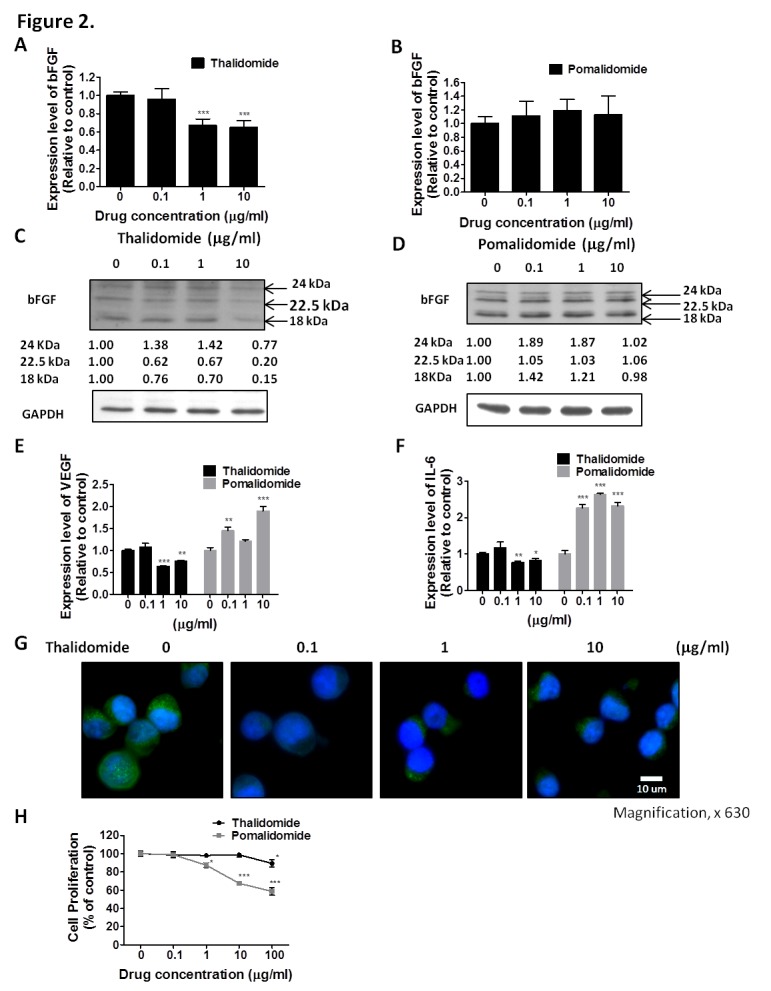
Effect of thalidomide or pomalidomide on bFGF, VEGF and IL-6 expression in U266 cells. U266 cells were treated with (A) thalidomide or (B) pomalidomide for 4 hours. The levels of bFGF mRNA were monitored by real-time PCR. The expression of GAPDH was used as an internal control. Protein extracts from U266 cells treated with (C) thalidomide or (D) pomalidomide for 4 hours were subjected to Western blot for analysis of bFGF protein level. GAPDH was the internal control. U266 cells were treated with thalidomide and pomalidomide for 4 hours and mRNA levels of (E) VEGF and (F) IL-6 were monitored by real-time PCR. (G) Thalidomide regulates bFGF expression and cellular distribution in U266 cells. Immunofluorescence detection of bFGF in U266 cells treated with thalidomide for 4 h. Cellular distribution of bFGF was studied by fluorescence microscopy. DNA was stained with H33258 as a nuclear marker. Magnification, × 630. (H) Viable cell counts using trypan blue. Cell viability of U266 cells treated with thalidomide and pomalidomide. U266 cells treated with the indicated concentrations (0, 0.1, 1 and 10 μg/mL) of thalidomide and pomalidomide for 72 hours were tested with trypan blue. Data were collected from at least 3 independent experiments. The results were expressed as the relative index of untreated control ± S.E.M. (*, P < 0.05, **, P < 0.01, ***, P < 0.001, Student's t-test).

To compare the therapeutic effect of thalidomide and pomalidomide, we used RPMI8226 and U266 cells, which are high-grade bFGF expressing human myeloma cell lines [32]. Our results showed that the bFGF mRNA levels in RPMI8226 (Figure 1A) cells and were markedly reduced after being treated with thalidomide for 4 h, even at 0.1 μg/ml and 1 μg/ml concentrations, which were lower than the reported serum concentrations (3–6 μg/ml) at most of the therapeutic doses in clinical patients [33]. However, the pomalidomide-treated cells did not present similar effects even at concentrations of 10 μg/ml (Figure 1B). Similar reduction of bFGF mRNA expression could be observed in U266 cells treated with thalidomide at 1 μg/ml (Figure 2A), but no effect on bFGF expression could be found in pomalidomide-treated cells even at the highest dose (Figure 2B). Further, Western blot analysis showed that thalidomide significantly diminished the expression of bFGF at 0.1 μg/ml (Figures 1C, 2C) but pomalidomide did not in RPMI8226 and U266 cells (Figures 1D, 2D). We studied expression level of bFGF in multiple myeloma cell from one multiple myeloma patient (newly diagnosed) by western blot. Our result showed that thalidomide significantly diminished the expression of bFGF at 0.1, 1 and 10 μg/ml (Figure S1). And we further used flow cytometric analysis to measure expression of bFGF. The thalidomide-treated RPMI8226 (expression level of bFGF was 31.72%, 22.53%, 20.75% and 11.81% at 0, 0.1, 1 and 10 μg/ml, respectively) (Figure S2A)and U266 MM cells (expression level of bFGF was 26.41%, 26.13%, 16.66 and 16.04% at 0, 0.1, 1 and 10 μg/ml, respectively) (Figure S2B).To validate the anti-angiogenic or anti-inflammatory effects of thalidomide and pomalidomide, we examined the VEGF mRNA levels and observed that thalidomide reduced VEGF mRNA at the concentrations of 0.1, 1 and 10 μg/ml, whereas pomalidomide significantly increased VEGF mRNA expression at the concentrations of 0.1, 1 and 10 μg/ml in RPMI8226 cells (Figure 1E). A similar trend was observed in U266 cells (Figure 2E). The expression of the inflammatory cytokine IL-6 was reduced by thalidomide at the concentrations of 1 and 10 μg/ml in RPMI8226 cells; pomalidomide also demonstrated an inhibitory effect at 0.1 and 1 μg/ml but not at the 10 μg/ml dose (Figure 1F). In U266 cells, thalidomide demonstrated an inhibitory effect on the IL-6 mRNA expression at 1 and 10 μg/ml; on the contrary, pomalidomide demonstrated stimulatory effects on the IL-6 mRNA (Figure 2F). And our immunofluorescence staining data showed that thalidomide down-regulated cellular bFGF content especially at the nuclear level in PMI8226 (Figures 1G, S3) and U266 (Figure 2G). We demonstrated the thalidomide regulates bFGF expression and cellular distribution in multiple myeloma Cells.

### Distinct Effects of Thalidomide and Pomalidomide on Cell Proliferation and Anchorage-Independent Growth

**Figure 3:**
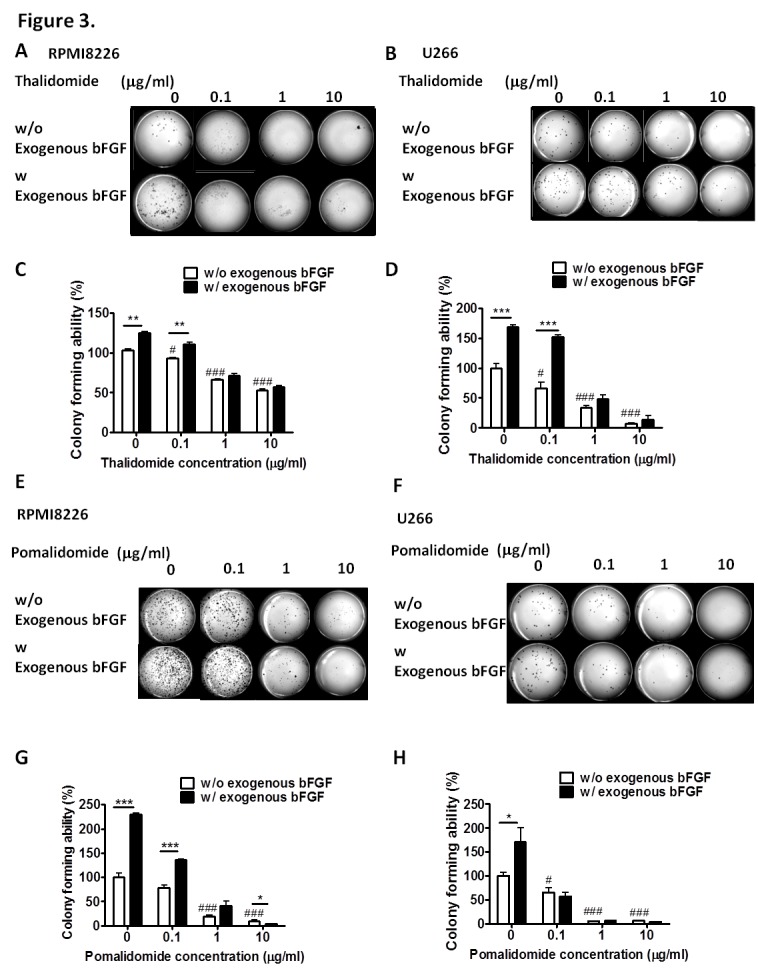
Effect of thalidomide or pomalidomide on AIG and its recovery in the presence of exogenous bFGF. The colony-forming ability of RPMI8226 and U266 cells seeded in culture medium containing 10% FCS and varied concentrations of (A, B) thalidomide and (E, F) pomalidomide plus 0.3% agar (size, >0.1 mm) were counted 14 d after treatment. Exogenous bFGF restores their colony-forming ability, and (C, D, G and H) are the quantification results. Data were collected from three independent experiments, with each experiment repeated three times. #, P < 0.05, ###, P< 0.001 versus control clone; **, P<0.01, ***, P < 0.001 versus without exogenous bFGF condition, Student's t test.

In the cell proliferation assay, only a high concentration (100 μg/ml) of thalidomide had a slight effect on the proliferation of RPMI8226 cells (cell viability was 84.09%, versus 100% in the control cells) (Figure 1H) and U266 MM cells (cell viability was 89.34%, versus 100% in the control cells) (Figure 2H). In contrast, pomalidomide-treated RPMI8226 (cell viability was 84.8% at 10 μg/ml and 75.35% at 100 μg/ml) and U266 MM cells (cell viability was 87.47%, 67.56% and 58.61% at 1, 10, and 100 μg/ml, respectively) demonstrated a dose-dependent inhibition of proliferation. An AIG assay conducted in soft agar demonstrated a significant reduction in the colony-forming ability of RPMI8226 or U266 MM cells in a dose-dependent manner following treatment with thalidomide (colony-forming ability was 92.7%. 66.15% and 52.7%) in RPMI8226 cells (Figures 3A, 3C) and U266 cells (colony-forming ability was 73.85%. 33.85% and 9.23%) (Figures 3B, 3D) or pomalidomide (colony-forming ability was 79.41%, 20.68% and 8.62%) in RPMI8226 cells (Figures 3E, 3G) and in U266 cells (colony-forming ability was 65.4%. 5.7% and 6.28%) (Figures 3F, 3H) at doses of 0.1, 1 and 10 μg/ml, respectively.

### Exogenous bFGF Can Restore the Inhibitory Effect of Thalidomide, but not Pomalidomide, on the AIG of the MM Cells

We next investigated the differential effect of bFGF on the AIG of MM cells; the aforementioned clones were incubated with recombinant 18 kDa LMW bFGF (20 ng) prior to the soft agar assay. The colony-forming ability could be restored by the addition of recombinant, 18 kDa bFGF (20 ng) at thalidomide concentrations of 0.1, 1 and 10 μg/ml (Figures 3A, 3B, 3C and 3D). The colony-forming ability, which was reduced in pomalidomide-treated cells at concentrations of 0.1, 1 and 10 μg/ml (Figures 3E, 3F, 3G and 3H), showed only a limited recovery following incubation with the recombinant 18 kDa bFGF (20 ng). These findings suggest that bFGF plays an important role in AIG and validate that thalidomide targets bFGF to reduce colony formation ability. The distinct effects observed in the presence of pomalidomide indicated the difference in mechanism between thalidomide and pomalidomide.

### The AIG of Multiple Myeloma is suppressed by the Knock-down of bFGF Expression Clones

**Figure 4:**
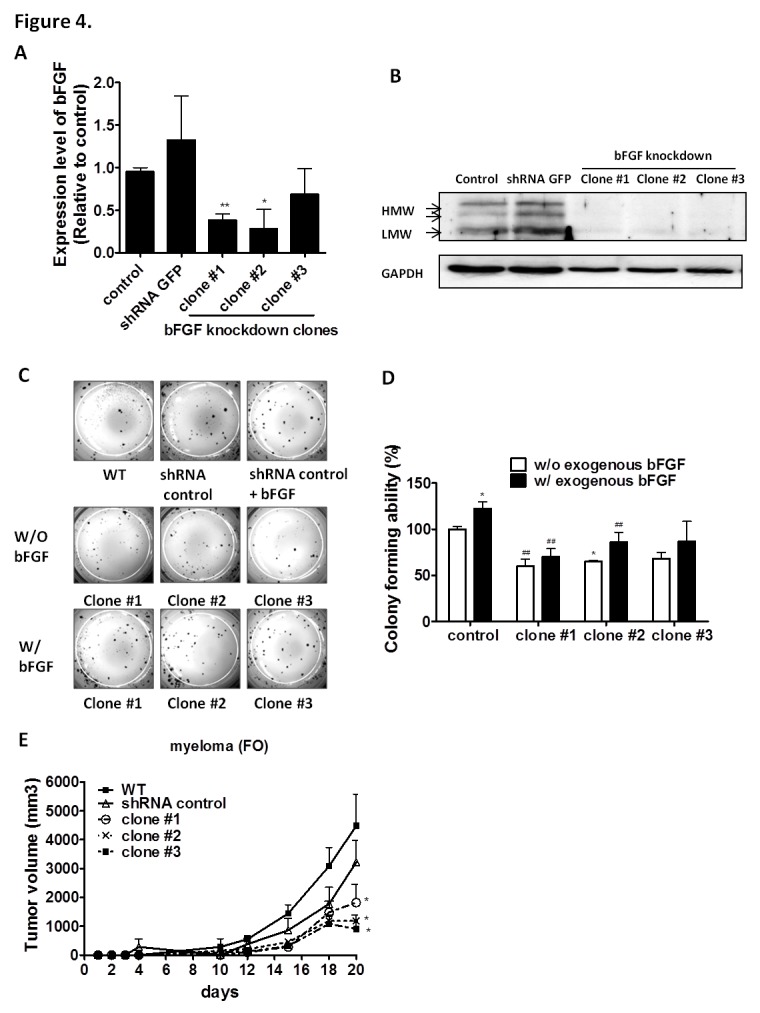
Down-regulating bFGF level is sufficient to inhibit the AIG of FO myeloma cells. (A) bFGF mRNA levels in bFGF knock-down clones and control cells were assayed by using real-time PCR or (B) bFGF protein levels in bFGF knock-down clones and control cells were analyzed by Western blot. (C) Down-regulating bFGF expression level in FO cells affects growth in soft agar. FO cells seeded in culture medium containing 10% FCS and 0.3% agar (size, >0.1 mm) were counted 14 d after treatment. Exogenous bFGF restores their colony-forming ability and (D) presents the quantification results. ##, P < 0.01 versus control clone, *, P< 0.05 versus no exogenous bFGF group, Student's t-test. (E) Tumor growth of control and bFGF knock-down clones. Tumor size was determined by using the following formula: 1/2 (length × width^2^). Data were represented as the mean values from one to six mice. Data were collected from three independent experiments, with each experiment repeated three times. *, P< 0.05, **, P< 0.01 versus the control clone, Student's t-test.

We next examined the tumorigenic potential of the bFGF knock-down FO (Figure 4) and RPMI8226 cells (Figure S4). Three different bFGF shRNAs (#1, #2, and #3) or the control shRNA were introduced into FO cells by lentiviral infection to generate three bFGF knock-down clones. We selected one clone for each shRNA and labeled them as clone #1, #2, and #3. We initially confirmed the knock-down efficiency of clone #1 (40.17%, p = 0.003), clone #2 (29.61%, p = 0.046) and clone #3 (71.88%, p = 0.0442) by QPCR at the mRNA (Figure 4A) and the protein level (Figure 4B). Further studies demonstrated that the bFGF knock-down clones possess lower colony-forming abilities in AIG (colony-forming abilities were 60%, 65.13% and 68.21%, respectively, for clones #1, #2, and #3, relative to the control) (Figures 4C, 4D). The colony-forming ability in LMW bFGF treated cells demonstrated only partial recovery (57.35%, 70.46% and 70.79%, versus exogenous bFGF control clone), indicating that HMW bFGF plays an important role in the AIG of MM cells. The measurement of tumor size is important in preclinical animal studies when assessing the response to cancer progression. One week following the implantation of pellets, 1×10^6^ control FO cells or FO cells expressing the knock-down bFGF clones #1, #2 or #3, were injected subcutaneously into the left and right flanks, respectively, in 100 μL PBS. Three weeks following the implantation of the tumor cells (tumor size 1090 – 4483 mm^3^), the six tumor volumes were determined *in vivo*; the tumor volume measured by a caliper correlated with the control volume. When we compared the WT with the shRNA control, clones #1, #2 and #3, the average tumor volumes were 4483.64 mm^3^±1100.16 mm^3^ (WT was 100%, mean ± S.E.M.), 3216 mm^3^±755.91 mm^3^ (shGFP was 71%, p = 0.365), 1821 mm^3^±602.22 mm^3^ (clone #1 was 40%, p = 0.039), 1184 mm^3^±203.91 mm^3^ (clone #2 was 26%, p = 0.015) and 1090 mm^3^±267.64 mm^3^ (clone # 3 was 24%, p = 0.010), respectively. Our results demonstrated that the bFGF knock-down clones possess lower tumorigenic abilities *in vivo* (Figure 4E).

### Thalidomide, but not Pomalidomide, Down-regulates bFGF Translation by Regulating its IRES Activity

**Figure 5:**
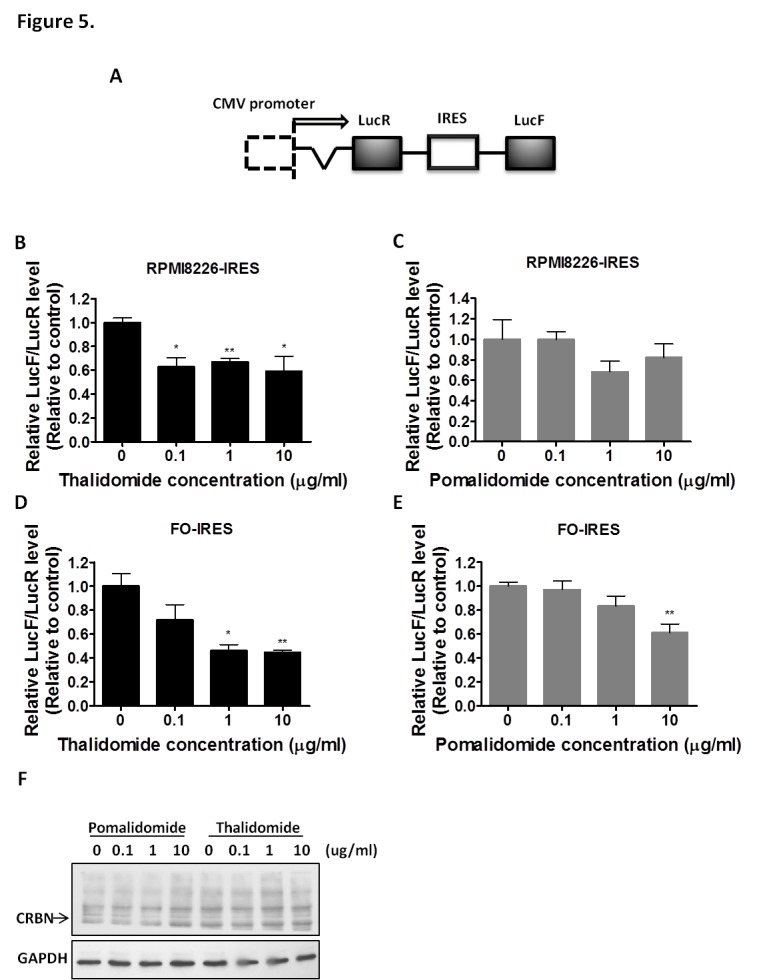
Comparison the effects of thalidomide or pomalidomide on bFGF-IRES activity and CRBN ubiquitination. (A) Schematic representation of HMW-IRES plasmids. (B) RPMI8226-HMW-IRES were treated with thalidomide. (C) RPMI8226-HMW-IRES were treated with pomalidomide. (D) FO-HMW-IRES cells treated with thalidomide and (E) FO-HMW-IRES cells treated with pomalidomide, were indicated as concentrations of drugs for 4 hours. Treated cells were lysed and luciferase activity was measured with Dual Luciferase Assay kits. IRES activity was determined by the ratio of Renilla luciferase (LucR) activity to firefly luciferase (LucF) activity. Columns, index of the ratio normalized with DMSO control (0.04%); bars, SD. *, P< 0.05, **, P< 0.01, Student's t test. (F) RPMI8226 cells were treated with the indicated concentrations of compounds for 4 hours. The endogenous CRBN was immunoblotted (IB) with anti-HRP antibody.

Our previous studies showed that thalidomide down-regulates bFGF HMW-IRES translation more than LMW-IRES bFGF by targeting the IRES region in gliomas [14]. Therefore, in the present study, we used the HMW bFGF IRES fragment that inserted into the bicistronic vector, as previously described, to generate the pHMW-IRES plasmids (Figure 5A). We used the IRES-dependent Dual Luciferase Reporter assay to compare the effective mechanism of thalidomide and pomalidomide. To explore the effects of thalidomide and pomalidomide on IRES activity (Firefly Luciferase), the signal was normalized to a Renilla Luciferase control. Our initial experiments demonstrated that HMW bFGF (22.5 kDa) was significantly down-regulated by thalidomide at 0.1 μg/ml (Figures 1C, 2C). Thus, when we treated thalidomide or pomalidomide for various time periods as indicated, the IRES activity was decreased as deduced via the activity ratio between Renilla luciferase and Firefly Luciferase, at 0.1, 1 and 10 μg/ml (IRES activity was 62.66%, 66.83% and 58.92%, respectively) for up to 4 hours in RPMI8226 cells (Figure 5B). However, we found that pomalidomide-treated RPMI8226 cells did not inhibit the IRES activity at 4 hours (Figure 5C). In FO cells, thalidomide altered the IRES activity at 1 and 10 μg/ml (46.18% and 44.56%, respectively) at 4 hours (Figure 5D), whereas in pomalidomide-treated FO cells, IRES activity was only moderately inhibited at the 10 μg/ml dose (IRES activity was 61.15%) at 4 hours (Figure 5E). Additionally, we compared the effect of thalidomide and pomalidomide on the endogenous ubiquitination of CRBN. A recent study using thalidomide affinity beads indicated that cereblon was a direct protein target for immunomodulatory and anti-proliferative activities of IMiDs [34]. In our study, thalidomide and pomalidomide did not affect the ubiquitination of cereblon in RPMI8226 MM cells (Figure 5F), indicating that the protein may not be the primary pharmacological target of thalidomide or pomalidomide. Whether CRBN is a secondary pathological target of thalidomide for its teratogenic effects needs further clarification.

### The Expression of bFGF is Concurrently Increased with PTCH1, the Hallmark of the Hedgehog Pathway, in MM Tumor Stem Cells upon AIG, which is the Hallmark of Tumorigenicity

**Figure 6:**
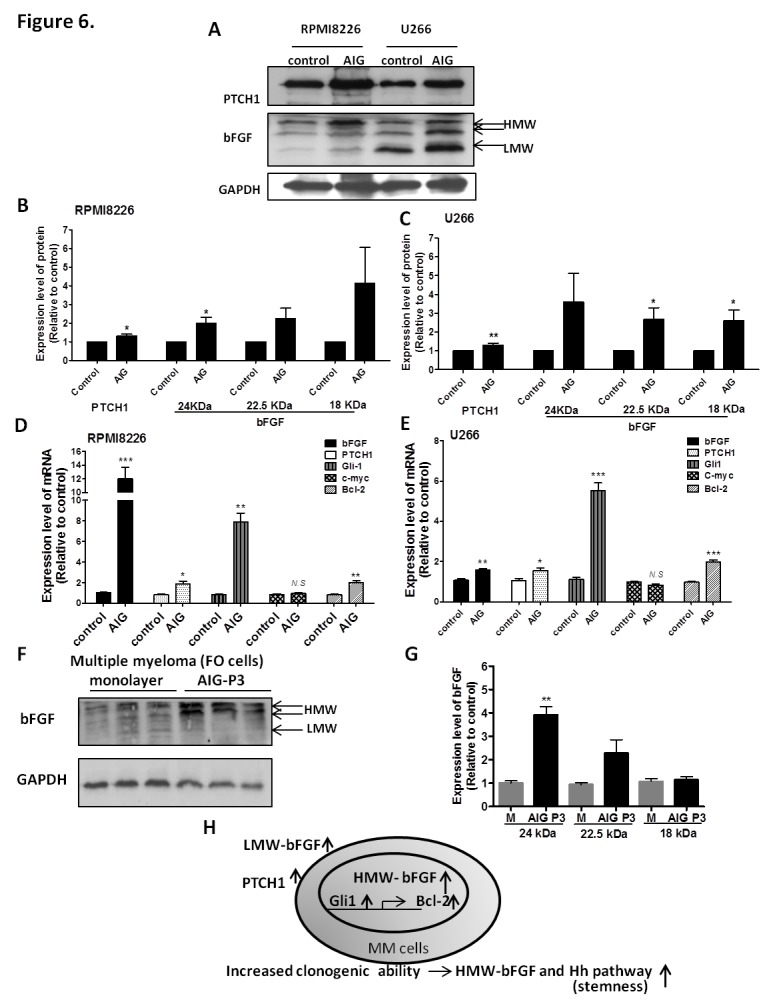
Expression of bFGF increases concurrent with PTCH1 upon AIG cultivation and passage in MM cells. (A) Protein extracts from RPMI8226 and U266 cells with AIG were subjected to western blot analysis for bFGF and PTCH1, with GAPDH as the internal control. (B, C) Quantification of bFGF protein levels in RPMI8226 cells by western blot and U266. (D, E) The mRNA expression of bFGF, PTCH1, Gli1, C-myc and Bcl-2 in clonogenic-RPMI8226 and U266 cells. (F) Protein extracts from FO cells with AIG were subjected to western blot analysis of bFGF. (G) Quantification results of Western blot. Data were collected from three independent experiments, with each experiment repeated six times. The results were expressed as the relative index of control or monolayer control ± S.E.M. (*, P< 0.05, **, P< 0.01, ***, P< 0.001, Student's t-test). (H) The model of our hypothesis shows potential mechanisms by which HMW-bFGF and Hedgehog pathway maintains myeloma stem cell features.

The AIG of cancer cells in vitro has proved to be a useful tool to enrich cancer stem-like cells [21], a subpopulation of tumor cells with the ability to undergo self-renewal and recapitulate the entire tumor population *in vitro* and *in vivo* [35]. In our studies, we compared culture medium cultivated (RPMI8226 and U266), monolayer (FO cells) and AIG of MM cells. We observed that the bFGF and PTCH1 mRNA levels in AIG-selected cells were increased 12.01-fold (p < 0.001) and 1.89-fold (p = 0.02) in RPMI8226 cells (Figure 6D) and mRNA expression of bFGF and PTCH1 were increased 1.59-fold (P = 0.004) and 1.55-fold (P = 0.04), respectively in U266 cells (Figure 6E). In RPMI8226 and U266 cells, the protein expression was increased 1.3-fold (p < 0.05) and 1.3-fold (p < 0.01), respectively (Figures 6A, 6B and 6C). Particularly, the HMW (24 kDa) bFGF expression increased 2.0-fold (p < 0.05), HMW (22.5 kDa) bFGF increased 2.2-fold (p > 0.05) and LMW bFGF increased 4.2-fold (p > 0.05) in RPMI8226 cells. In U266 cells, our data showed that the protein expression of HMW (24 kDa) bFGF increased 3.6-fold (p > 0.05), HMW (22.5 kDa) bFGF increased 2.7-fold (p < 0.05), and LMW bFGF increased 2.6-fold (p < 0.05) (Figures 6B, 6C), and to investigate whether hedgehog pathway activates PTCH1 signaling through regulation of Gli1 target genes. Our results showed the Gli1and Bcl-2 mRNA levels in AIG-selected cells were increased 7.88-fold (p = 0.001) and 1.99-fold (p = 0.01), respectively in RPMI8226 cells (Figure 6D). The mRNA expression of Gli1 and Bcl-2 were increased 5.5-fold and 1.99-fold, respectively in U266 cells (Figure 6E). In FO cells, our data showed that the protein expression in generations one to three of AIG-selected cells, HMW (24 kDa) bFGF was increased 4.1-fold (p < 0.01), HMW (22.5 kDa) bFGF was increased 2.3-fold (p > 0.05), and LMW bFGF was increased 1.1-fold (p > 0.05) (Figures 6F, 6G). Previous studies have indicated that the Hh pathway activation is heterogeneous across the spectrum of MM tumor stem cells [36]. We also compared a recent reported MM tumor stem marker PTCH1 and found that the expression of bFGF and PTCH1 are concurrently increased. It activates Hh pathways, possibly by regulating the transcription of Gli1 and other stem cell genes, thereby maintaining the stem cell state of these cells (Figure 6H).

## DISCUSSION

Although some mechanisms have been proposed to explain the activity of thalidomide and IMiDs in MM, such as anti-angiogenic, anti-cell proliferative, or immunomodulatory effects, the precise cellular targets and molecular mechanisms remain unclear [37]. Our results showed that the mRNA and protein levels of bFGF are inhibited by low concentration thalidomide in MM cell lines (Figures 1A, 1C, 2A and 2C) but not by pomalidomide (Figures 1B, 1D, 2B and 2D). Interestingly, we found that thalidomide did not inhibit MM cell proliferation, whereas pomalidomide showed a significant inhibitory effect on these MM cells (Figures 1G, 2G). Drucker *et al.*[38] reported that relatively high concentrations of thalidomide (> 25 μg/ml) could down-regulate the transcription for genes with GC-rich promoters. However, the concentration used in these studies was much higher than the clinically achievable serum concentrations of patients. IMiDs were generated by the chemical modification of thalidomide for the development of anti-cell proliferation drugs. For example, recent results reported that the IMiDs showed higher cytotoxic effects than thalidomide in a number of MM cells [34]. Both lenalidomide and pomalidomide, which can inhibit MM cell growth at a low concentration of 0.1 μM, are potent cytotoxic drugs compared with thalidomide, which needs a 1000-fold higher dose to reach its cytotoxic effect on MM cells. However, although many anti-cancer agents were developed as cytotoxic drugs, the majority of patients relapse as a result of metastasis.

Thalidomide treatment was initiated in MM because this condition correlates with prominent bone-marrow vascularization, which is associated with poor prognosis. In addition, plasma levels of various pro-angiogenic molecules, such as basic fibroblast growth factor or vascular endothelial growth factor, are increased in patients with active MM. Therefore, anti-angiogenic drugs, such as thalidomide, are viable therapeutic options [39]. In addition, these IMiDs also display immunomodulatory and anti-apoptotic effects, but little is known about their primary mode of therapeutic action in patients with cancer. However, Liu *et al.* confirmed that IMiDs were independent of a specific cytotoxic effect in colorectal carcinoma cells, but associated with intracellular signaling [40]. The mechanism of action of IMiDs is complex and it probably includes different molecular targets dependent on different concentrations or dosages. So far, at least 30 hypotheses about thalidomide's mechanism of action have been proposed [41]. However, despite years of research into the clinical uses and its precise cellular targets and mechanism of action is incomplete.

Our results demonstrated that thalidomide could inhibit the clonogenic ability of MM cells at the concentration of 0.1 μg/ml, which was lower than the clinically achievable plasma concentrations in MM patients (Figures 3A, 3B). Our previous report also showed that 0.1 μg/ml thalidomide could inhibit the colony-forming ability of glioma cells. In the current study, we highlight a non-cytotoxic anticancer drug target of thalidomide, the IRES of bFGF, and provide the mechanistic rationale for developing IMiDs as more potent anti-cancer therapeutics in MM patients with fewer side effects. Using thalidomide as a tool, we identified the GC-rich promoter coding sequences of the genes as a novel target for cancer chemotherapy and identified the molecular basis for drug development in cancer. Our study highlighted a non-cytotoxic anticancer drug target for thalidomide, the IRES of bFGF, and provide the mechanistic rationale for developing IMiDs as anti-MM therapeutics. Our finding also offer a direct approach to enhance the efficacy and reduce the side effects of thalidomide by slow release technology, and might contribute to therapy for MM patients in the near future.

Recently, spheroid culture gene expression profiles have been shown to reflect clinical expression profiles more accurately than those observed in monolayer cultures [42]. The HMW bFGF may play an extremely important role in the nucleus in regulating tumor cell survival and metastasis [18]. Our results showed that the bFGF knock-down significantly reduced AIG (Figures 4C, 4D), indicating that bFGF plays a pivotal role in AIG but not in cell proliferation. Notably, the down-regulation of bFGF translation via inhibition of HMW-IRES is specific based on the results obtained from the dual luciferase assays (Figures 5B, 5C, 5D and 5E). So far, thalidomide is the first drug demonstrated to target HMW-IRES, both in solid tumors and MM, especially at the lower concentration of 0.1μg/ml, which is even lower than the serum concentration obtained in thalidomide-treated patients. However, thalidomide was reported to readily undergo rapid hydrolysis to chirally stable teratogenic metabolites [43, 44]. Our studies emphasize that the lower concentration of thalidomide could effectively target the IRES of bFGF, which could be achieved by using sustained-release technology platforms to enhance the therapeutic effect or to decrease the unwanted side effects of thalidomide.

CRBN was recently identified as a primary target of thalidomide teratogenicity [45] using a newly developed affinity purification technique. The studies demonstrated that CRBN directly binds to thalidomide affinity beads and is linked to the teratogenic effect of thalidomide in zebrafish. This complex regulates DNA repair, replication and transcription, and inhibition of it by IMiDs may causes teratogenic effect, which highlight it may play a crucial role in embryonic limb development. Lopez-Girona recently reported that cereblon is a direct protein target of lenalidomide and pomalidomide, and with cytotoxic effect on MM cells even at the concentration of 0.1 μM [34]. Instead, our data showed thalidomide did not show significant inhibitory effect on proliferation even at 100 μg/ml. Besides, in their cereblon-overexpression HEK 293 cells (not MM cells), thalidomide only at 30μM can be observed the inhibition of CRBN autoubiquitination. However, 30μM is not the achievable serum concentration in thalidomide treated MM patients. In our study, thalidomide at 0.1 μg/ml targeted to IRES bFGF involving clonogenicity of MM cells as the primary pharmacological target, Instead, thalidomide and pomalidomide even at 10μg/ml concentration did not affect the ubiquitination of cereblon in RPMI8226 MM cells (Figure 5F), indicating that this protein may not be the primary pharmacological target of thalidomide or pomalidomide in MM patients. Some reports indicated the CRBN expression decreases in MM patients that developed resistance to IMiDs therapy [46]. However, these papers showed the high CRBN expression in RPMI8226 [47] or JJN3 [46] cell lines, but these two cell lines were defined as IMiDs-resistant cell lines [48]. Although this report indicated the CRBN expression is correlated in refractory patients, but their data showed the CRBN expression of three patients did not decrease, even one of patient was increased from these results, we suggested that CRBN may not be the primary pharmacological target in IMiDs, but can be the teratogenic target of IMIDs. According to recent reports of Kronke *et al.* [49] and Lu *et al.* [47], IMiDs have a toxic effect on multiple myeloma by causing the degradation of two transcription factors, Ikaros and Aiolos through the CRBN. We suggested that these two transcription factors are cytotoxic targets of IMIDs, and CRBN might be the teratogenic target of IMIDs. Instead, our findings highlight a non-cytotoxic primary target for stemness of MM which may constitute the majority of tumor cells.

Neoplastic plasma cells are the hallmark of MM. However, despite these therapies, MM remains largely incurable [50]. Disease relapse suggests that the cells responsible for tumor re-growth are relatively drug resistant [51], but a full understanding of the cell type responsible for MM growth remains unclear. Early studies examining a murine model of multiple myeloma suggested only a minority of cells were capable of clonogenic growth [52]. Salmon and Hamburger [21] found that the cloning efficiency of primary MM specimens was 0.001 to 0.1%. To date, it has remained unclear whether these clonogenic cells are distinct from the plasma cells that constitute the majority of tumor cells. Our results showed that the expression of bFGF increased upon AIG (Figure 6), indicating that stemness might be one of the characteristics for MM cells with clonogenic activity.

Cancer stem cells (CSCs) have been proposed as the initiators of tumorigenesis and the seeds of metastases [53]. They are a subpopulation of tumor cells with the ability to undergo self-renewal and recapitulate the entire tumor population *in vitro* and *in vivo* [35]. Only a few cancer cells can successfully survive the multistep metastatic process. Although the precise mechanism of these cancer cells remains undefined, some properties of CSCs, such as AIG of cells that display stem cell properties, mediates metastasis [24]. In addition to the capability of self-renewal, CSCs have the ability to initiate distant metastases that resemble the primary tumors and are resistant to conventional chemotherapy/radiotherapy, implicating them in tumor growth and recurrence [54]. Our results demonstrated that clonogenic cells possess stem cell-like characters (Figure 6). Interestingly, our data showed that the expressions of bFGF are increased upon AIG. It was reported that the cells expressing the HMW bFGF protein presented more drug resistance and gene amplification potential compared with LMW-bFGF expressing cells in a soft agar assay [55]. Our results showed that a low concentration of thalidomide 0.1 μg/ml, but not pomalidomide, could inhibit the expression of bFGF through IRES upon AIG, indicating a non-cytotoxic mechanism for the inhibition of cancer stem-like cells.

Lenalidomide was developed as IMiDs for its immunomodulatory activity up to 50,000-fold more potent at TNFα inhibition in vitro compared with thalidomide, also much more potent than thalidomide in its ability to co-stimulate T cells [37]. However, the clinical efficacy was not parallel with its immunomodulatory activity. According to recent review article of clinical trials of MM, the drugs used as a single agent for its anti-MM property were compared [56]. When these active drugs for MM sorted by best response rate, thalidomide was 59% [57], and higher than pomalidomide's 54% [58]. When sorted by average response rate, thalidomide was still higher than pomalidomide and lenalidomide [56].

Taken together, our study highlighted that the IRES of bFGF is the non-cytotoxic primary molecular target of thalidomide and should be considered the target for the development of IMiDs in multiple myeloma. Moreover, by using the sustained-release technology to protect thalidomide stability, it would be worthwhile to direct future studies towards developing thalidomide or its analogues as anti-metastasis drug via inhibition of IRES of bFGF or stemness.

## MATERIALS AND METHODS

### Cell Culture and Transient and Stable Transfection

RPMI8226, U266 and FO cells were obtained from the Bioresource Collection and Research Center, Taiwan and cultured in RPMI 1640 or DMEM supplemented with 10% heat-inactivated fetal bovine serum and antibiotics. The cells were transfected using Lipofectamine transfection Reagent (Invitrogen, Paisley, UK) according to the manufacturer's protocol. The three different bFGF shRNAs (#1, #2, and #3) or the control shRNA were introduced into multiple myeloma cells by lentivirus infection to generate three bFGF knock-down clones; the sequences are presented in Supplementary Table S2. These clones effectively down-regulated the expression of endogenous bFGF (Figures 4A, 4B, S4A, S4B and S4C); however, the doubling time was similar to that of the control clone (data not shown).

### Thalidomide and Pomalidomide Treatment

Thalidomide (a gift from TYY Biopharm) and Pomalidomide (Sigma) were dissolved in DMSO to generate a stock concentration of 25 mg/ml, and further dilutions to the desired drug concentration were conducted only in the culture medium. The maximum final concentration of DMSO in all cultures was 0.04%. For the pre-incubation test, thalidomide and pomalidomide were diluted to the indicated concentrations in the culture medium alone and incubated in the CO_2_ incubator for 4 h before being added to the cells.

### RNA Isolation and Real-time PCR

Total RNA was extracted using the RNA-Bee RNA isolation solvent (Tel-Test); 5 μg RNA was then converted into cDNA using the Moloney murine leukemia virus reverse transcriptase (Promega). Real-time PCR primers targeting human glyceraldehyde-3-phosphate dehydrogenase (GAPDH) and bFGF were designed using the Primer Express software (Applied Biosystems); the sequences are presented in Supplementary Table S1. The ABI Prism 7500 Sequence Detection System and the SYBR Green Master Mix kit (both from Applied Biosystems) were used for the real-time PCR analysis of the reverse-transcribed cDNA samples. The expression level of human GAPDH was used as an internal reference. Relative gene expression levels were calculated with the 2^-ΔΔCT^ method [14].

### Immunofluorescence

Cells grown on glass coverslips were fixed in PBS containing 4% paraformaldehyde for 15 min and then permeabilized with 0.01% Triton X-100 for 30 min at room temperature. The cells were subsequently treated with 0.5 μg of a polyclonal rabbit anti-human bFGF peptide, amino acids 40 to 63, antibody (Abcam) for 30 min at room temperature. Cells were then washed and stained with Alexa Fluor® 488 goat anti-rabbit secondary antibodies (Life Technologies) for another 30 min. The cells were then washed and visualized with a fluorescence microscope (Leica DM 6000B Mycrosystems, Wetzlar, Germany).

### Dual LuciferaseAssay

The luciferase assays were conducted using a Dual Luciferase Reporter Assay System according to the protocol specified by the manufacturer (Promega), and the luciferase activity was quantified by scintillation counting in a Victor2 1420 Multilabel Counter (Wallac, Perkin-Elmer). The relative IRES activity was represented as an index of the ratio of Renilla luciferase to firefly luciferase, normalized to the untreated control. All experiments were performed in triplicate and repeated at least twice [29].

### Cell Proliferation Assay

For proliferation assays, cells were treated with or without thalidomide or pomalidomide for 72 h. Then, the cell number and viability were measured by Trypan blue assay.

### Colony Formation Assay

A total of 2,000 cells were plated in 0.3 ml of RPMI 1640 with 0.3% agarose and 10% fetal bovine serum; cells were layered on top of 0.5% agarose at 0.3 ml per well in a 24-well plate of DMEM with 10% fetal bovine serum. After 2 wk, cells were stained with 3-(4,5-dimethylthiazol-2-yl)-2,5- diphenyltetrazolium bromide (Sigma-Aldrich), plates were photographed, and colony numbers were counted. The results represent the mean of three individual experiments [14, 21].

### Immunobloting

Cell lysates were prepared using a RIPA lysis buffer (1x RIPA lysis buffer: 50mM Tris-HCl, pH7.5; 10mM EDTA; 1% NP-40; 0.1% SDS; 150nM NaCl; 1mM PMSF) and tumor conditioned media were concentrated by lyophilizing. For Immunoblot analyses, equal amounts (50 μg) of protein samples were resolved on a 15% polyacrylamide gel, and transferred to PVDF membrane before probing with anti-bFGF polyclonal rabbit antibody (ab16828, Abcam) at 1:1000 dilutions. Anti-GAPDH (ab9482, Abcam) at 1:10000 dilutions was also used as the internal control. Protein was stained using HRP-conjugated anti-IgG secondary antibody, and ECL for the detection (Amersham) [14].

### Plasmid Construction

Genomic DNA was purified from U-87 cells. Approximately 500 ng genomic DNA was used as a template and the amplification was performed using an ABI 2700 Thermo cycler and Taq polymerase (Genet Bio). The PCR fragments were amplified according to the following described previously [14]. The primers used to amplify the bFGF promoter HMW-IRES are shown in Supplementary Table S1. For plasmid construction, PCR products were T/A cloned into a pGEMTeasy vector (Promega).

### Colony Passage

Clonogenicity of cells with AIG was determined by a colony-forming cell (CFC) assay. The FO, RPMI8226 and U266 cells (9 × 10^4^) were prepared in a 100-mm culture dish. Suspension cells were maintained in 7 ml complete growth medium, 2X base medium, 1% methylcellulose and 20% FBS. The colonies in methylcellulose were collected by gentle centrifugation (1000 rpm for 15 minutes), dissociated with 0.25% trypsin–EDTA and mechanically disrupted with a fire-polished Pasteur pipette. The cell pellet was resuspended in 1 ml of fresh medium and designated as *P1.* The recovered cells were subsequently seeded in a new 100-mm petri dish at a density of 9 × 10^4^ cells per dish, with complete growth medium supplemented with 20% FCS and 1% methylcellulose, and cultured in the incubator for 14 days. The *P2* or *P3* cells were obtained by repeating all of the above steps [23].

### *In Vivo* Murine Tumorigenicity Model

The FO cells (mice myeloma cell line) and the FO-bFGF knock-down clones (clone #1, #2 and #3) were cultured in DMEM supplemented with 10% fetal calf serum and 1% penicillin-streptomycin, in 5% CO_2_ at 37°C. The six 7-wk-old BALB/c mice were s.c. implanted with control knock-down cells (1 × 10^6^ per mouse) in the left flank and then randomly divided into three groups. The tumor size was calculated by external caliper measurements every other day. Three weeks after implantation of tumor cells (tumor size 1090 – 4483 mm^3^), the volumes of the 6 tumors were determined using an external caliper.

## SUPPLEMENTAL FIGURES, TABLES AND MATERIALS AND METHODS


