# Supplementary material for: Internal ribosome entry site of bFGF is the target of thalidomide for IMiDs development in multiple myeloma
Source: Genes Cancer. 2014 Mar;5(3-4):127–41. doi: 10.18632/genesandcancer.11 (PMC4091528; doi:10.18632/genesandcancer.11)
Supplement: Supplementary file 1 [file ganc-05-127-s001.pdf]

Internal ribosome entry site of bFGF is the target of thalidomide for IMiDs development in multiple myeloma

Materials and Methods

Patient samples

Bone marrow specimens were obtained from multiple myeloma patients at the Taipei Veterans General Hospital. Live, mononuclear cells (with 30% plasma cells) were isolated from bone marrow aspirates by Ficoll-Hypaque density gradient centrifugation. Cell lysates were prepared using a RIPA lysis buffer (1x RIPA lysis buffer: 50mM Tris-HCl, pH7.5; 10mM EDTA; 1% NP-40; 0.1% SDS; 150nM NaCl; 1mM PMSF). For Immunoblot analyses, equal amounts (50 µg) of protein samples were resolved on a 12% polyacrylamide gel, and transferred to PVDF membrane before probing with anti-bFGF polyclonal rabbit antibody (sc-7911, SANTA CRUZ BIOTECHNOLOGY, INC.,) at 1:1000 dilutions. Anti-GAPDH (ab9482, Abcam) at 1:10000 dilutions was also used as the internal control. Protein was stained using HRP-conjugated anti-IgG secondary antibody, and ECL for the detection (Amersham) (Mei *et al.*, 2008).

Detection of bFGF, by flow cytometry

After treating with thalidomide. Harvest cells and determine total number present. Wash twice in PBS. Following staining, wash cells once in PBS and discard supernatant cells were collected in 1.5 ml eppendorff. Cells were then permeabilized with 0.5 % saponin contained PBS for 30 min at room temperature. Cells were subsequently treated with 0.5 µg of a polyclonal rabbit anti human bFGF antibody (ab16828, Abcam) for 30 min at room temperature. Then, washed, and stained with secondary antibodies for another 30 min (1: 250; goat anti-rabbit IgG Alexa 488). Flow cytometry analysis was performed on a FACS calibur, and data were analyzed using CellQuest software (Becton–Dickinson, BD Biosciences San Jose, CA).

Table S1 List of primer sequence

GAPDH		
	Forward	5’-AATGTCACCGTTGTCCAGTTG-3’
	Reverse	5’- GTGGCTGGGGCTCTACTTC-3’
bFGF		
	Forward	5’- ATCAAAGGAGTGTGTGCTAACC-3’
	Reverse	5’- ACTGCCCAGTTCGTTTCAGTG-3’
PTCH1		
	Forward	5’-TTCCAGTTAATGACTCCCAAGCAAATG-3’
	Reverse	5’-GCGACACTCTGATGAACCACCTC-3’
VEGF		
	Forward	5’-GCACATAGGAGAGATGAGCTTC-3’
	Reverse	5’-CCACAGGGACGGGATTTCTTG-3’
IL-6		
	Forward	5’-GTACATCCTCGACGGCATCTC-3’
	Reverse	5’-AGCCATCTTTGGAAGGTTCAG-3’
HMW-IRES		
	Forward	5’ -CTC CTG ACG CGT CAG GAG GGA GGA GGA CTG G-3’
	Reverse	5’ -CTC ACA ACG GGT TGT GAG GGT CGC TCT TCT C-3’

Table S2 Sequence of bFGF shRNA

Sequence ID	Target gene	Target sequence
#1	bFGF	ACTACAATACTTACCGGTCAA
#2	bFGF	GTTACGGATGAGTGTTTCTTT
#3	bFGF	GCAGTCATAAACAGAAGAATA
control	shGFP	ACGTCTATATCA ATGGCCGACA

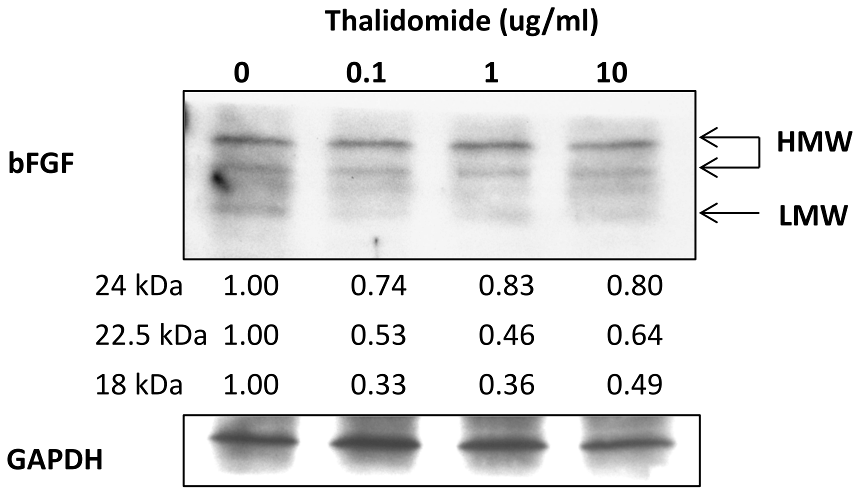

Figure S1: Effect of thalidomide on bFGF expression in patient's sample. Protein extracts from patient's mononuclear cells treated with for 4 hours were subjected to Western blot for analysis of bFGF protein level. GAPDH is the internal control.

A

RPMI8226

Thalidomdie (μg/ml)

0 0.1 1 10

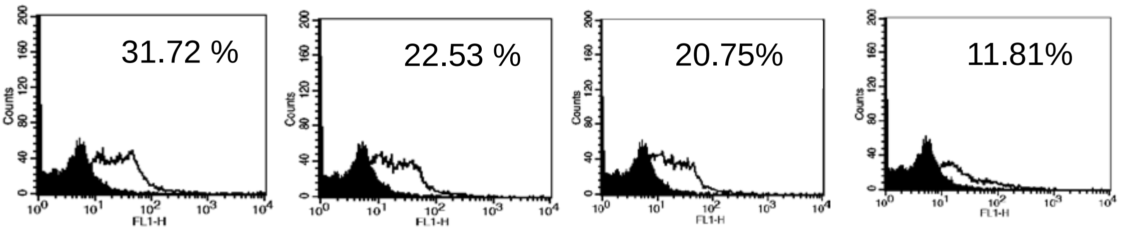

B

U266

Thalidomide (μg/ml)

0 0.1 1 10

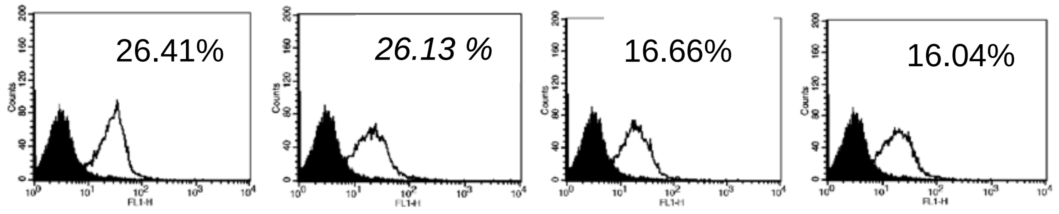

Figure S2: Fluorescence histogram from analysis of bFGF expressing multiple myeloma cells. Flow cytometry analysis of bFGF expression (black lines) by cells treated with thalidomide. Black line, specific antibody; filled black histograms, isotype-matched control antibody. Data are representative of three independent experiments.

PMI8226

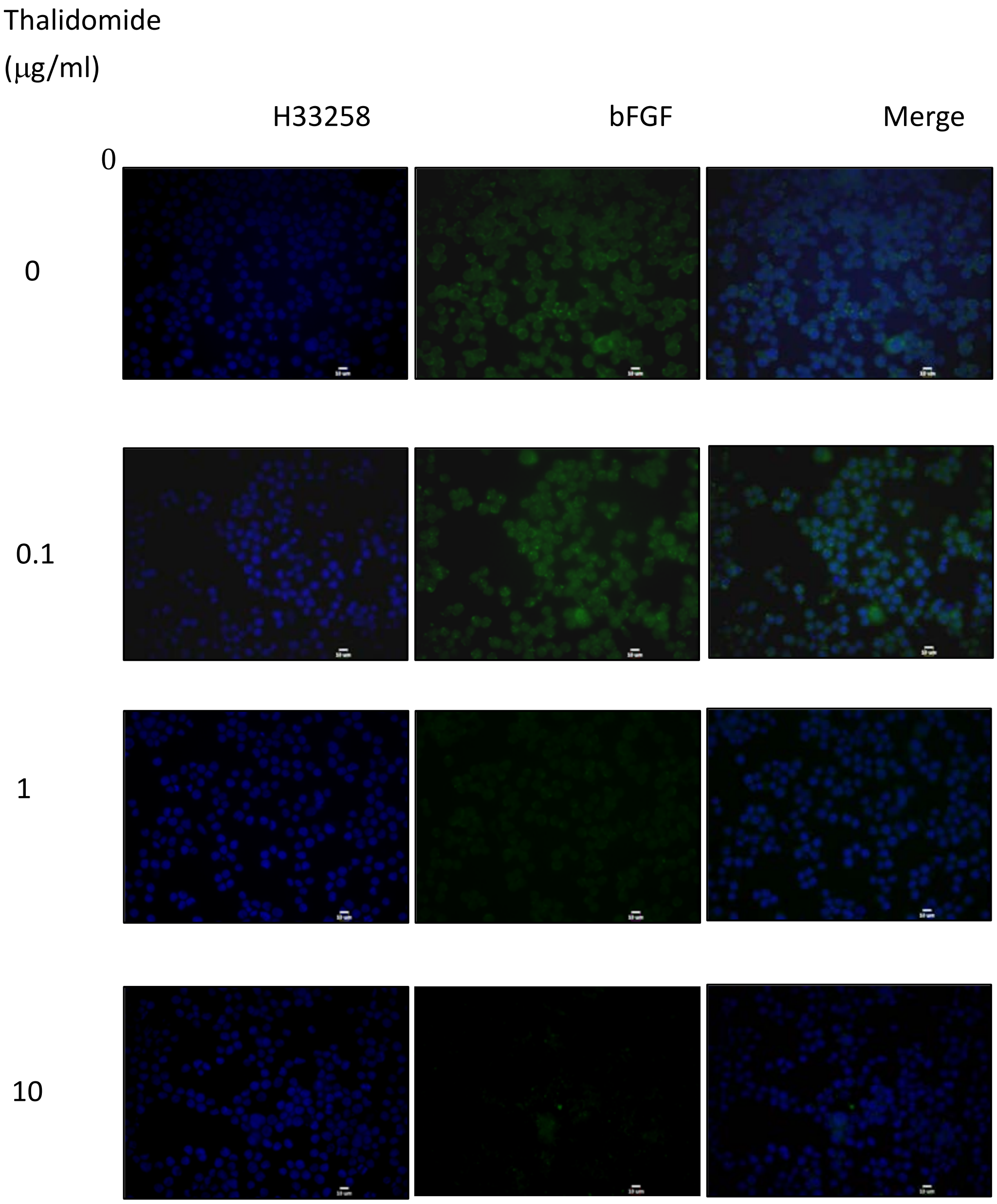

Maganification X 400 Scale bar, 10 µm

Figure S3: Immunofluorescence analysis of cellular bFGF distribution of RPMI8226 cells after treating thalidomide. The first panel represented bFGF distribution stained by rabbit anti-bFGF polyclonal antibody. Second panel were labeled with secondary Alexa Fluor 488 antibodies (green channel) and cell nuclei labeled with H33258 (blue channel). The third panel is the merged images of the first and second panel.

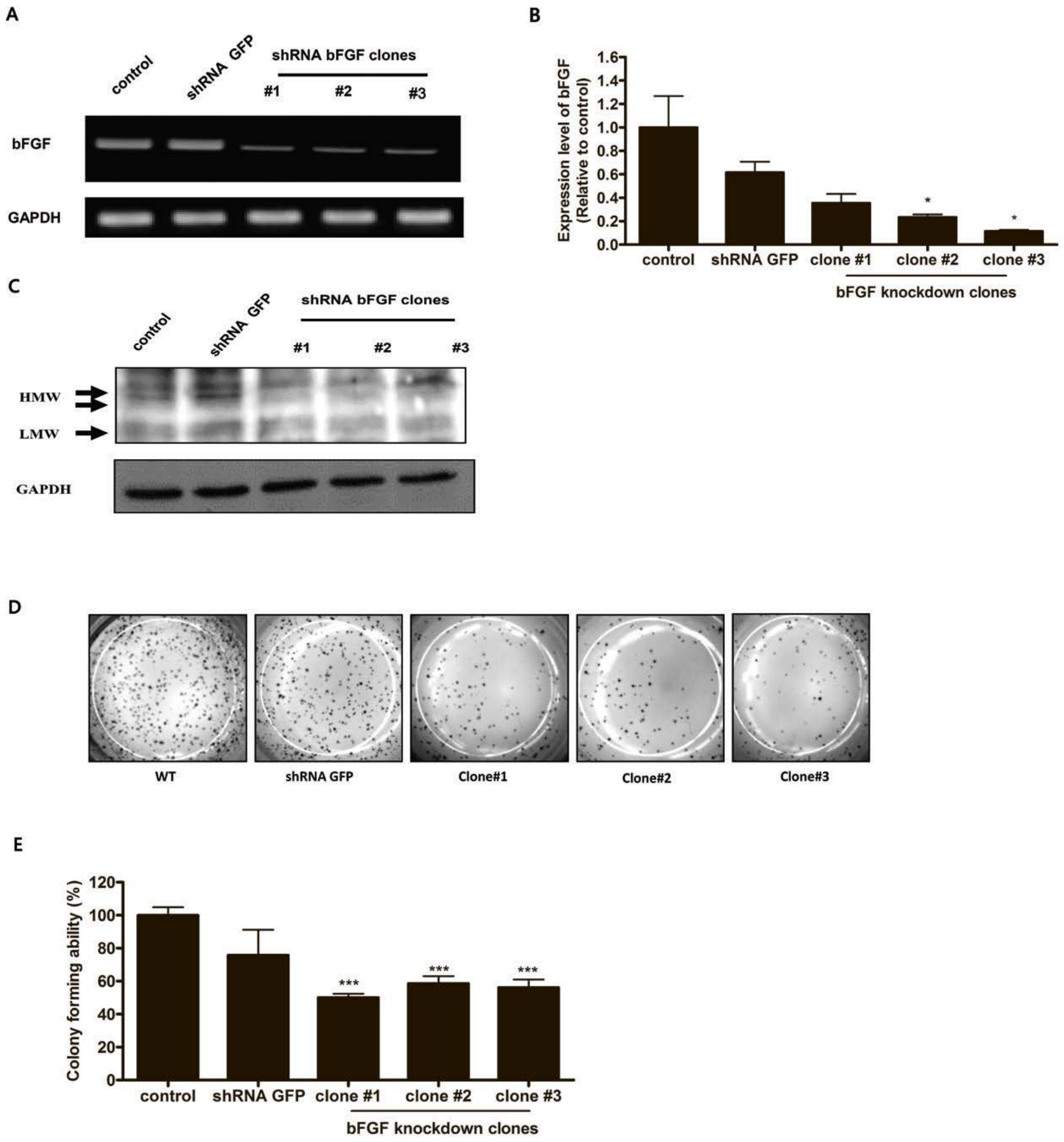

Figure S4 : Down-regulating bFGF expression is sufficient to decrease the AIG of RPMI8226 cells. bFGF mRNA levels in bFGF knock-down clones and control cells were assayed with (A) RT-PCR or (B) Q-PCR. (C) bFGF protein levels in bFGF knock-down clones and control cells were analyzed by Western blot. (D) Colony-forming ability (size, >0.1 mm) was measured 14 d later. Data were collected from three independent experiments, with each experiment repeated six times. (E) is the quantification result. Data were collected from three independent experiments, with each experiment repeated six times. *, P<0.05, ***, P < 0.001 versus control clone, Student’s t-test.
